# A road data assets revenue allocation model based on a modified Shapley value approach considering contribution evaluation

**DOI:** 10.1038/s41598-024-55819-7

**Published:** 2024-03-02

**Authors:** Shiwei Li, Lei Chu, Jisen Wang, Yuzhao Zhang

**Affiliations:** 1 School of Traffic and Transportation, Lanzhou Jiaotong University, Lanzhou, 730070 China; 2 Key Laboratory of Railway Industry on Plateau Railway Transportation Intelligent Management and Control, Lanzhou, 730070 China

**Keywords:** Road data asset, Two-layer revenue allocation, Index evaluation, Modified Shapley value, Rough set, Computational science, Scientific data

## Abstract

This paper constructs a two-layer road data asset revenue allocation model based on a modified Shapley value approach. The first layer allocates revenue to three roles in the data value realization process: the original data collectors, the data processors, and the data product producers. It fully considers and appropriately adjusts the revenue allocation to each role based on data risk factors. The second layer determines the correction factors for different roles to distribute revenue among the participants within those roles. Finally, the revenue values of the participants within each role are synthesized to obtain a consolidated revenue distribution for each participant. Compared to the traditional Shapley value method, this model establishes a revenue allocation evaluation index system, uses entropy weighting and rough set theory to determine the weights, and adopts a fuzzy comprehensive evaluation and numerical analysis to assess the degree of contribution of participants. It fully accounts for differences in both the qualitative and quantitative contributions of participants, enabling a fairer and more reasonable distribution of revenues. This study provides new perspectives and methodologies for the benefit distribution mechanism in road data assets, which aid in promoting the market-based use of road data assets, and it serves as an important reference for the application of data assetization in the road transportation industry.

## Introduction

Data collection and processing have become more convenient and intelligent with the development of information technologies like the Internet of Things and artificial intelligence. As a result, all industries now have massive amounts of data. Data, as a new production factor, harbors immense potential value. However, disorganized data is worthless. Its true value can only be realized by transforming raw data into standardized, complete, and accurate data resources through data governance. The term "data asset" was first coined by Richard Peterson in 1974. Later, the 2012 World Economic Forum report considered data as a new category of economic asset. In 2021, the China State Administration for Market Regulation (CSAMR), together with the China Standardization Administration (CSA), issued a national standard document (GB/T 40685-2021) defining data assets as measurable and legally sourced data resources capable of generating economic and social value^1^.

Since its inception, the transport industry has been generating massive amounts of data, which are huge in scale and high in precision and quality. Road transport, as an important part of the transport industry, generates a large amount of road data during its operation. The data assets formed after sorting and processing will provide support for the optimal design of the road network, the emergency response of the road network, and the optimization of vehicle routes to comprehensively guarantee the construction of smart roads. Therefore, accelerating the circulation and trading of road data assets to enable effective excavation and commercialization of data value has become crucial.

In contrast to the banking, Internet, pharmaceutical, and other industries that can complete the entire data value chain within the enterprise, the resources and technologies mastered by enterprise subjects in the road transport industry vary greatly, making it difficult to complete the entire process of data generation, collection, and processing independently. Table 1 compares several key characteristics of data asset evaluation across four major industries: road transport, banking, internet, and pharmaceutical.

**Table 1:** Comparison of data asset evaluation characteristics across industries.

Industry	Data value chain	Data ownership	Computational resources	Regulatory factors
Road transport	Fragmented data collection, information silos	Fragmented corporate data ownership	Limited computational resources	Strict industry regulation
Banking	Unified data infrastructure	Banks own user data	Abundant cloud computing resources	Strict financial regulation
Internet	Abundant user behavior data	Platforms' user data	Massive computational capabilities	Relatively lax regulation
Pharmaceutical	Reliant on experimental data	Proprietary R&D data	Require massive computational resources	High regulatory standards

The road transport industry contends with unique challenges in asset evaluation compared to other sectors for several reasons. Firstly, road transport data remains fragmented across proprietary silos in the value chain, whereas banking has established unified data systems and infrastructure. Secondly, road transport corporate data is highly dispersed unlike consolidated user data assets held by banks and internet platforms. Pharmaceutical firms also possess proprietary R&D datasets^2^. Thirdly, road transport has relatively limited computational capabilities contrasted with the abundant scalable cloud resources of banks and internet companies. Pharmaceuticals likewise require significant computing power for R&D^2^.

Road transport faces more stringent regulatory oversight than sectors like internet companies, which contributes to its disadvantages in data and analytics capabilities. However, regulations alone do not fully explain these gaps. Banking and pharmaceuticals also operate under strict supervision, yet boast stronger data and computational resources than transport. To address the data development challenges in the road transport industry, it is imperative to propose innovative solutions within the regulatory framework.

Overcoming limitations in data access and analytics is critical for realizing the enormous value of road transport data. However, the substantial differences in resources and technologies possessed by different road transport companies often result in mismatches between data ownership and data processing capabilities, highlighting the need for cross-enterprise collaboration. From the perspective of the data value chain, collaborative enterprises can be divided into three roles: original data collectors, data processors, and data product producers. Given the scope, complexity, and diversity of road data and participants, it is vital to investigate fair and reasonable revenue distribution mechanisms.

At the moment, the market-based trading of road data assets is in its early stages, with little research on the revenue allocation mechanism of data assets and nearly no special studies on the distribution mechanism of road data assets. This paper develops a two-layer road data asset revenue allocation model based on the modified Shapley value method. The first layer allocates revenues to three types of roles, namely, original data collectors, data processors, and data product producers, and corrects them using data risk factors. The second layer determines the respective correction factors for different roles to realize the distribution of revenues to participating enterprises under different roles, and finally synthesizes the revenues of the participating enterprises under each role to obtain the final revenue of each participating enterprise. The purpose of this paper is to conduct an exploratory investigation on the subject of revenue allocation of road data assets to fill gaps in related research in this field. The results of the study will, to a certain extent, promote the marketed circulation and application of road data assets and help the development of smart road construction.

The main contributions of this paper are as follows: (1) We expand the research in the field of road data assets from the perspective of revenue allocation, applying the Shapley value method in cooperative game theory to achieve unique and fair revenue allocation so that relevant participants in the data value chain can get due reward. (2) For situations with many cross-cutting participants in road data assets, we divide them into three roles based on the process of realizing data value: original data collectors, data processors, and data product producers. We then construct a two-layer revenue allocation model from roles to participating enterprises. (3) Considering the limitations of the traditional Shapley value method, we establish a revenue distribution evaluation index system for revision, using entropy weighting and rough set theory to determine index weights. We adopt fuzzy evaluation and numerical analysis to comprehensively calculate participants' contributions across qualitative and quantitative aspects. This paper constructs a fair and reasonable revenue allocation model for road data assets, providing methods and suggestions for market-based trading of such assets with certain reference values for promoting data assetization in road transport.

This paper is structured as follows: In Section “Literature review”, we review the literature related to data asset trading, road digitization, and revenue allocation methods. In Section “Model building”, we analyze the participating subjects of road data assets and divide them into three roles. We construct a two-layer revenue allocation model for the roles and participating enterprises within them and correct the initial allocation of the traditional Shapley value through a revenue evaluation index system. In Section “Case study”, we verify the proposed model's effectiveness through a case study of road data assets. Finally, in Section “Conclusions”, we summarize the research process for this paper.

## Literature review

### Road digitalization

As a technological advancement, digitalization has permeated various aspects of the economy and society. The academics describe the essence of digitalization from the perspectives of product and value creation, stating that it facilitates the transition from one-way to two-way product design, enabling interactive and configurable products, and promoting the co-creation of the product value.

Among them, "road digitalization" is based on collecting data through various types of sensing equipment, relying on the multi-network convergence of communication facilities to transmit data, and through intelligent analysis and processing, to achieve highway control guidance, intelligent decision-making, personalized services, etc. This effectively improves the safety level of the transportation system, traffic efficiency, and management effectiveness.

Some scholars have already conducted research in the field of road digitalization. Singh et al.^3^ extensively examined the significance of road digitalization from various aspects such as intelligent lighting systems, smart emergency management systems, and renewable energy. They also described the architectures of intelligent lighting systems and smart emergency management systems. Lu et al.^4^ constructed a real-time digital model of traffic scenes based on vision, which supports the development of digital twins of road traffic to a certain extent. With the support of these digitalization technologies for roads, the application value of road data assets can be fully explored.

Road data assets refer to various digital resources related to roads, including dynamic data such as technical indicators, traffic flow, weather conditions, and vehicle routes. These data can be used for traffic monitoring and prediction^5^, signal control, and road condition feedback^6^. For example, the Beijing Municipal Commission of Transport has opened up traffic-related data to travel service platforms such as Amap, Baidu Maps, and Meituan, enabling these platforms to provide new features such as bus occupancy rate query, comprehensive comparison of travel plans, and estimated travel time, which comprehensively improves the level of traffic and travel services.

The digitalization of roads provides a data foundation for road data assets through various sensing devices that collect dynamic road data. The application of road data assets elevates roads from static construction to "networked, sensed, and intelligent" dynamic management, which is the key foundation for smart transportation development. Data asset trading provides an opportunity for the open sharing of road data, creating revenue for relevant transportation enterprises and further enhancing the value and influence of road digitization. This process advances the scientific, intelligent, and efficient development of road management.

### Data assets trading

The transition of data from being perceived as mere objects to being regarded as valuable assets signifies its significant contribution to economic development^7^. There are two main ways to realize the economic value of data assets: one is to bring economic benefits indirectly by optimizing business processes and assisting decision-making within the enterprise; the other is to sell the data assets directly to the outside world in the data trading market so that more enterprises can benefit from them and fully activate the value of these data assets.

Data transactions are typically facilitated by three parties: data consumers, data providers, and data markets. Data providers package and submit their data to the data market, which then matches the appropriate data providers with the needs of data consumers. Finally, data providers and consumers interact to finalize the transaction^8^. Acting as intermediaries, data markets primarily provide services such as data legality examination, quality assessment, and value evaluation.

Europe and the United States have explored data trading earlier, and currently, active big data trading platforms include Dawex (France), Streamr (Sweden), Advaneo (UK), Otonomo (Israel), and so on. In 2015, China began implementing its big data strategy and established the first domestic big data exchange institution, the Guizhou Big Data Exchange. In 2019, China further proposed participating in the distribution of data as a factor of production, and data trading organizations were established one after another in Beijing, Shanghai, and Shenzhen, marking that data trading has entered a period of rapid development in China.

Data products sold by data trading platforms mainly include different forms such as data packages, API interfaces, and data analysis reports. Differences in the form of data products affect the formulation of pricing strategies. Existing data pricing strategies can be classified into six categories: free data, usage-based pricing, package pricing, uniform pricing, freemium pricing, and two-part pricing combining bundle and uniform pricing^9^. Data trading cannot be realized unless the precise selling price of the data set is established. Data pricing needs to meet the requirements of revenue maximization, fairness, arbitrage-free pricing, computational efficiency, etc.^9^, and more scholars have explored and researched data pricing methods. Liang et al.^10^ explored the factors affecting the data price based on the feature price model in terms of the data object, the data seller, and the data buyer. Tian et al.^11^ focused on the data seller as the main entity and designed optimal contract mechanisms considering privacy protection in various market scenarios, aiming to achieve individual rationality and incentive compatibility. Oh et al.^12^ designed a competitive Internet of Things (IoT) data trading environment consisting of data providers, data brokers, data service providers, and data consumers. They also proposed a unified method for pricing data sets to compare the competitiveness of different data brokers.

In addition to considering the transaction scenario and market supply and demand conditions, data pricing also entails focusing on the inherent value and potential contributions of the data. Some scholars have conducted research on the valuation of data assets from the perspective of data intrinsic characteristics. Yu et al.^13^ proposed a data pricing model that takes into account data quality and versioning strategies, enabling data quality assessment and market segmentation. Liao et al.^14^ quantified user privacy choices and constructed a multi-scenario data property bilateral trading model. Chellappa et al.^15^ conducted a detailed analysis of version control strategies for data products and derived the optimal version of data products along with corresponding prices.

In the process of data transactions, various technical means need to be applied to protect data security and the rights of data rights holders. Currently, technologies such as privacy computing^16^, blockchain^17^, and digital watermarking^18,19^ can support the platform's data protection efforts, set up the platform's data protection system, and consider data security and compliance when conducting transactions.

In summary, as a new type of strategic resource, data assets have data trading as one of the important means to realize their commercial value. Promoting the transaction and utilization of data assets is an important development trend nowadays, which can create value for data participants. Data trading platforms should activate the value of data while maintaining data security and complying with regulatory requirements to promote the orderly circulation of data resources. Revenue allocation is the primary task following data asset transactions, serving as a key motivator to stimulate the active participation of enterprises. A fair and equitable revenue allocation mechanism can promote and incentivize deep open sharing and the value creation of data assets.

### Revenue allocation methods

For the revenue allocation of data assets, there is a lack of mature allocation methods, and the Shapley value method based on cooperative game theory is widely used in revenue allocation problems in various fields. The Shapley value method can achieve a unique and fair distribution of asset benefits by calculating the marginal contributions of each participant in different combination scenarios. Luo et al.^20^ proposed a rapid calculation method of accurate Shapley value under the independent utility for multi-source datasets, but this method only considers the data owner's benefit allocation and does not cover other participants in the data value chain.

The basic Shapley value method treats all participants as equal in status and distributes benefits based solely on the average marginal contribution, without considering the differentiated contributions of the participants. To overcome this shortcoming and make the revenue allocation reasonable, many scholars have tried to introduce factors such as input cost, risk-taking, and urgent demand based on the Shapley value method to reflect the asymmetric contributions of the participants. Wang et al.^21^ established a modified Shapley value method based on cloud gravity, taking into account risk, inputs, and service quality, and applied it to the revenue allocation of a private charging pile-sharing project, which significantly improves the effect of multi-party cooperation. Yang et al.^22^ constructed a modified Shapley value-based integrated energy system revenue-sharing model based on operational risk factors, which can reflect the actual operational risk and the degree of contribution of participants. Zheng et al.^23^ introduced five non-cooperative and cooperative models for a remanufacturing closed-loop supply chain. They considered the bargaining power of alliances as the game's bottom line and proposed a method of variable-weighted Shapley value to achieve profit distribution in the supply chain.

The roles and tasks performed by different parties in a cooperative alliance differ, and the Shapley value based on contribution alone cannot fully account for other key factors, such as resource input and risk-taking by the participants, making the benefit distribution scheme unfair to some extent. Therefore, to achieve reasonable benefit distribution and stable cooperation in the data asset value chain, the basic Shapley value method needs to be improved by selecting appropriate modifying factors for the specific conditions of the value chain to take into account the contributions, inputs, risks, and other factors of the participants in a fair manner.

In conclusion, for the problem of data asset revenue allocation, the method based on the Shapley value method has the advantage of being uniquely fair, but it also has the defect of considering only the average contribution and ignoring the differentiated contribution. To achieve fairness and efficiency in revenue allocation, it is necessary to follow the principle of fair distribution of the Shapley value method, fully consider the differentiated characteristics of each participant in the value chain, and use appropriate modifying factors to design an improved scheme that can take into account both the fairness of revenue allocation and the stability of alliance cooperation. The study of revenue distribution of road data assets by modifying the Shapley value method can achieve fair and reasonable revenue sharing among the participants, stimulate data sharing, cooperation, and innovation, and further optimize road construction and management decisions.

### Ethical declaration

The research described in this paper focuses on developing a revenue allocation model for road data assets using a modified Shapley value approach. The data referenced is simulated and does not contain any real or private information about individuals or organizations. All data of the revenue distribution model discussed are entirely fictitious and fabricated for the sole purpose of demonstrating the proposed approach. No actual road assets or transportation systems data has been accessed or analyzed without appropriate consent. This research does not involve the collection of any confidential data or infringement on privacy rights. The study does not aim to cause harm or unfairly benefit any entities. As this is theoretical research for academic purposes only, it does not have any current real-world implications. The research methodology and proposed model strive to maintain ethical standards, avoid conflicts of interest, and uphold principles of fairness and integrity.

## Model building

### Players

This paper divides the process of realizing the value of highway data assets into three key stages: original data collection, data processing, and data product development. Based on this process, the main stakeholders in distributing revenues from highway data assets can be divided into three roles: First, the original data collectors, namely the initial holders of road data, who complete the original collection of road data and own these data; second, the data processors, who add value to the original data through cleansing, integration, analysis, mining, and other means; third, the data product producers, who utilize the processed data for product design, development, and operation, realizing the full commercial value of the data assets.

#### Original data collectors

They obtain revenues by collecting road original data, which are primarily generated from enterprises' activities in road construction and operation management. For example, traffic volume and speed data collected by road authorities through fixed monitoring devices; toll station traffic volume and toll data acquired by toll road operators; real-time traffic conditions and route data collected by map service companies using navigation devices; vehicle status and road condition data gathered by automakers through onboard devices.

#### Data processors

They obtain revenues by processing the lawfully acquired original road data using methods like standardization, cleansing, integration, mining, etc. This process requires building road data warehouses, establishing analytical models, and discovering correlations in the data to extract value from the data. Since the original road data has a large volume but low-value density, it cannot be directly used for knowledge discovery and decision support. Only by improving data quality and discovering potential value through processing can more valuable highway data assets formed. For example, road research institutes analyze and integrate data collected by road authorities to support transportation planning; intelligent connected vehicle companies develop data models, leveraging data gathered by onboard devices to forecast traffic volume; mobility service platforms fuse user feedback with driving data to enhance traffic condition judgment and vehicle dispatching capabilities.

#### Data product producers

Based on the processed datasets, they obtain revenues by developing data products with practical value, marketing, and maintaining these products. Major data product formats include data packages, API interfaces, data analytical reports, etc. These road data products require continuous development and maintenance by data product operators. For example, road monitoring systems developed by transportation authorities for government users to improve road safety; ETC systems developed by new infrastructure operators, providing services like toll payment inquiries; usage-based auto insurance products developed by insurance companies using vehicle driving data to charge premiums based on mileage.

It should be emphasized that since road data rights can be separated and shared, different interests can be allocated to different stakeholders as needed, and the same participant may also simultaneously take on roles in multiple stages of the data value chain. For example, some transportation operators are responsible for both original data collection and participation in data processing and product design. Therefore, the distribution of revenues from road data assets should be reasonably determined based on the contributions made by each participant at different stages.

### Evaluation indicator system for revenue allocation

The traditional Shapley value method only allocates revenues based on marginal contributions, while participants in the same role may have significant differences in costs, risks, and other aspects. These differences need to be fully considered in the revenue distribution process. In addition, generating road data assets requires the participation of original data collectors, data processors, and data product producers. In practice, some participants may simultaneously take on multiple roles. The revenue distribution needs to comprehensively consider their contributions across different roles.

To address these issues, this paper proposes a two-layer allocation mechanism based on the traditional Shapley value method to reasonably distribute revenues from road data assets. The first layer determines the revenue shares for the three roles based on their contributions in the value chain; the second layer further distributes the revenues of each role to the actual participants. Compared to the Shapley value method, which only considers marginal contributions, this two-layer allocation mechanism is more comprehensive and reasonable, as it additionally takes into full consideration the differences in costs and risk sharing among different participants, as well as the contributions of the same participant under different roles. By considering both role contributions and participant efforts, the two-layer allocation mechanism achieves fair and effective revenue distribution.

#### First layer: role revenue allocation

During the lifecycle of road data assets, all participants face various risks, and those taking on more risks expect higher returns. Therefore, this paper takes the data risk factor as a correction factor for role benefit allocation. According to the sources, data risks are divided into external risks and internal risks. External risks mainly include policy risks and legal risks, as changes in relevant policies and the enactment of laws regarding data assets can significantly impact participants' operations. Internal risks refer to those arising from equipment failures, data security, and other factors during the generation of road data assets, which can be prevented and controlled.

#### Second layer: revenue allocation among participants of the same role

Based on the characteristics of the three roles—original data collectors, data processors, and data product producers—specific indicators that influence revenue distribution among participants within each role are constructed respectively.

For original data collectors, their contribution lies in planning the collection of high-value original data. This paper employs three indicators—construction cost, data demand, and data characteristics—to adjust the revenue of the original data collection participants. Construction cost covers the major costs involved in the production process of original data, including sub-indicators of data planning, data collection, and data storage. Data demand is assessed by examining the scarcity and application value of the data in the market and is further divided into two sub-indicators: demand extent and scarcity level. Considering the large volume but low-value density of original road data, data coverage and timeliness of updates are included as sub-indicators under data characteristics.

For the data processors, their contribution lies in transforming the original data into high-quality data with application value. Two indicators—data cleansing and data analysis—can be adopted to evaluate the contribution of data processing participants. Data cleansing is considered the fundamental task in data processing, and its effectiveness can be assessed using the sub-indicators of data volume and data quality. As the core of extracting data value, data analysis can be evaluated based on the quality of analysis methods and analysis utility as sub-indicators.

For the data product producers, their contribution lies in developing products and services for end users based on processed data, as well as managing the operation of the products. Two indicators—product development and product maintenance—can be employed to assess the contribution of data product producers. Product development is evaluated based on the workload and difficulty coefficient as sub-indicators, while product maintenance is evaluated based on stability and update frequency as sub-indicators.

**Figure 1:**
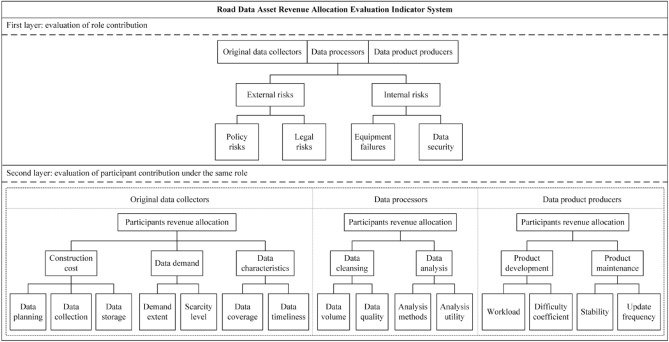
Evaluation indicator system for road data asset revenue allocation.

Overall, the evaluation indicator system for road data asset revenue allocation constructed in this paper comprehensively considers the contributions of different roles and participants in the road data asset value chain. The specific indicators are illustrated in Fig. 1, and Table 2 provides detailed explanations of the definitions, calculation methods, and value ranges for each indicator.

**Table 2:** Description of evaluation indicators for road data asset revenue allocation.

Layer	Indicator	Indicator definitions	Indicator value
Evaluation of role contribution (the first layer)	External risks	The external environment constraints and influences on road data collection and use reflect the role's contribution in identifying, assessing, and responding to external risks	Subjective indicator: determined through fuzzy comprehensive evaluation method
	*Policy risks*	It requires close attention to changes and adjustments in relevant policies to promptly take corresponding measures for risk management and response	The value is taken from expert evaluation scores
	*Legal risks*	It requires compliance with relevant laws, regulations, and policy requirements to ensure the legality, security, and compliance of data	
	Internal risks	It is usually caused by inadequacies in aspects such as management, technology, and decision-making, reflecting the role's contribution in managing and controlling internal risks	Subjective indicator: determined through fuzzy comprehensive evaluation method
	*Equipment failure*	It requires measures such as regular equipment inspection and maintenance, establishing comprehensive data backup and recovery mechanisms, etc. to reduce the likelihood of equipment failure	The values are taken from expert evaluation scores
	*Data security*	It requires measures such as establishing network security protection systems, strengthening access control and identity authentication mechanisms, etc. to enhance data security	
Evaluation of participant contribution under the role of the original data collectors (the second layer)	Construction cost	It reflects the cost contribution of the data collection participant, which needs to be estimated based on the specific situation	Objective indicator: determined through accounting cost calculation
	*Data planning*	The costs spent on market research and requirements analysis before data collection, to determine specific needs and functional requirements	The values are in monetary amounts
	*Data collection*	The costs incurred for purchasing and setting up the collection equipment and associated supporting facilities	
	*Data storage*	The costs sustained to establish appropriate data management and storage systems to effectively manage and store the collected data	
	Data demand	It describes the importance and difficulty of obtaining data to reflect the level of supply and demand of data in the market	Subjective indicator: determined through fuzzy comprehensive evaluation method
	*Demand level*	It describes the importance and urgency of data requirements	The values are taken from expert evaluation scores
	*Scarcity level*	It describes the availability and supply level of data	
	Data characteristics	The quantitative information that describes the characteristics and attributes of a dataset reflects the value of the dataset itself	Objective indicators: determined through numerical analysis
	*Data coverage*	It delineates the scope of highway data collection, encompassing temporal and spatial coverage parameters	The values are time span and geographical scopes
	*Data timeliness*	It depicts the freshness of the data, as timely updated highway data can furnish accurate, real-time traffic information	The value is update frequency
Evaluation of participant contribution under the role of the data processors (the second layer)	Data cleansing	The objective is to ensure the quality and accuracy of data, which is a fundamental task in data processing work	Objective indicators: determined through numerical analysis
	*Data volume*	It describes the workload of data processing	The value is the number of data records
	*Data quality*	It is measured in terms of the degree of improvement in the completeness, validity, and consistency of the data compared to the original data	The value is the percentage of total data that meets the criteria
	Data analysis	It discovers correlations, trends, patterns, etc. in the data to provide understanding and decision support for the problem	Subjective indicator: determined through fuzzy comprehensive evaluation method
	*Analysis methods*	Techniques used to understand and interpret data, and appropriate analytical methods facilitate the discovery of potential information in the data	The value is taken from expert evaluation scores
	*Analysis utility*	It measures the practical value of data analysis results in decision-making and problem-solving by evaluating the effectiveness of providing insights that guide decision-making and improve performance	
Evaluation of participant contribution under the role of the data product producers (the second layer)	Product development	Transforming road data into practical tools and solutions is the primary task for data product producers	Objective indicators: determined through numerical analysis
	*Workload*	It measures the time and resources expended on product development	The value is the number of valid codes
	*Difficulty coefficient*	It measures the difficulty and complexity of product development	The value is 0–1 based on compared with the evaluation criteria
	Product maintenance	During the product operation stage, support, bug fixing, and improvements are carried out to ensure the continuous and reliable operation of the product and adapt to user needs and market changes	Objective indicators: determined through numerical analysis
	*Stability*	The running status of the product needs to be monitored, and any faults or errors that occur need to be addressed and fixed promptly	The value is the average number of stable days per month for the product
	*Update frequency*	As market demands are updated and change, there is a need for continuous improvements to the product's functions and features to provide greater value and user experience	The value is the average interval time of product innovation

### Conventional Shapley value

The Shapley value method is a cooperative game approach used to solve the problem of profit distribution in multi-party cooperation. It determines the allocation of profits for each participant based on their marginal contributions. It is known for its characteristics of simple model construction, easy solvability, and unique solutions, allowing for a balance between efficiency and fairness in the distribution process.

#### First layer: role revenue allocation

Suppose in a road data asset revenue distribution, the three roles of original data collectors, data processors, and data product producers are represented by the set $R = \{ 1,2,3\}$. For any subset (representing any combination of roles in the role set), there exists a real-valued function $v(s)$, satisfying:

$$v(\emptyset ) = 0$$

1 $$v(s_{1} \cup s_{2} ) \ge v(s_{1} ) + v(s_{2} ),\begin{array}{*{20}c} {} & {s_{1} \cap s_{2} } \\ \end{array} = \emptyset ,\begin{array}{*{20}c} {} & {s_{1} ,s_{2} \subset R} \\ \end{array}$$

$[R,v]$ is termed the cooperation strategy of the three roles, and $v$ represents the characteristic function of the cooperation strategy.

$x_{i}$ denotes the fraction of the maximum revenue $v(R)$ from the road data asset that role $i$ receives. Based on the cooperative strategies $[R,v]$, the income distribution among the three roles is represented by $x = (x_{1} ,x_{2} ,x_{3} )$. A successful cooperative strategy must satisfy the following conditions:

$$\begin{array}{*{20}c} {x_{1} + x_{2} + x_{3} = v(R)} & {i = 1,2,3} \\ \end{array}$$

2 $$x_{i} \ge v(i),\begin{array}{*{20}c} {} & {i = 1,2,3} \\ \end{array}$$

where $\varphi_{i} (v)$ represents the distribution obtained by role $i$ under the cooperative strategy $[R,v]$. The Shapley value for each role's income distribution under the cooperative strategies is given by $\Phi (v) = (\varphi_{1} (v),\varphi_{2} (v),\varphi_{3} (v))$:

$$\varphi_{i} (v) = \sum\limits_{{s \in s_{i} }} {w(\left| s \right|)[v(s) - v(s\backslash i)]\begin{array}{*{20}c} {} & {i = 1,2,3} \\ \end{array} }$$

$$w(\left| s \right|) = \frac{(3 - \left| s \right|)!(\left| s \right| - 1)!}{{3!}}$$

3 $$\varphi_{1} (v) + \varphi_{2} (v) + \varphi_{3} (v) = v(R)$$

where $s_{i}$ is a set containing all subsets of $R$ that include role $i$, $\left| s \right|$ is the number of elements in subset $s$, $w(\left| s \right|)$ is the weighting factor, $v(s)$ is the revenue for subset $s$, and $v(s\backslash i)$ represents the revenue that can be obtained by removing role $i$ from subset $s$.

Therefore, the Shapley value method is applied to evaluate the contributions of the three roles in the road data asset, and the calculations for revenue allocation are presented in Table 3.

**Table 3:** Road data asset role revenue allocation.

(a) Revenue allocation for the original data collectors				
*s*_1_	{1}	{1,2}	{1,3}	{1,2,3}
$v(s)$	$v(1)$	$v(1,2)$	$v(1,3)$	$v(1,2,3)$
$v(s\backslash 1)$	0	$v(2)$	$v(3)$	$v(2,3)$
$v(s) - v(s\backslash 1)$	$v(1)$	$v(1,2) - v(2)$	$v(1,3) - v(3)$	$v(1,2,3) - v(2,3)$
$\left| s \right|$	1	2	2	3
$w(\left| s \right|)$	1/3	1/6	1/6	1/3
$w(\left| s \right|)[v(s) - v(s\backslash 1)]$	$v(1)/3$	$[v(1,2) - v(2)]/6$	$[v(1,3) - v(3)]/6$	$[v(1,2,3) - v(2,3)]/3$
$\varphi_{1} (v)$	$v(1)/3 + [v(1,2) - v(2)]/6 + [v(1,3) - v(3)]/6 + [v(1,2,3) - v(2,3)]/3$			

(b) Revenue allocation for the data processors				
*s*_2_	{2}	{1,2}	{2,3}	{1,2,3}
$v(s)$	$v(2)$	$v(1,2)$	$v(2,3)$	$v(1,2,3)$
$v(s\backslash 2)$	0	$v(1)$	$v(3)$	$v(1,3)$
$v(s) - v(s\backslash 2)$	$v(2)$	$v(1,2) - v(1)$	$v(2,3) - v(3)$	$v(1,2,3) - v(1,3)$
$\left| s \right|$	1	2	2	3
$w(\left| s \right|)$	1/3	1/6	1/6	1/3
$w(\left| s \right|)[v(s) - v(s\backslash 2)]$	$v(2)/3$	$[v(1,2) - v(1)]/6$	$[v(2,3) - v(3)]/6$	$[v(1,2,3) - v(1,3)]/3$
$\varphi_{2} (v)$	$v(2)/3 + [v(1,2) - v(1)]/6 + [v(2,3) - v(3)]/6 + [v(1,2,3) - v(1,3)]/3$			

(c) Revenue allocation for the data product producers				
*s*_3_	{3}	{1,3}	{2,3}	{1,2,3}
$v(s)$	$v(3)$	$v(1,3)$	$v(2,3)$	$v(1,2,3)$
$v(s\backslash 3)$	0	$v(1)$	$v(2)$	$v(1,2)$
$v(s) - v(s\backslash 3)$	$v(3)$	$v(1,3) - v(1)$	$v(2,3) - v(2)$	$v(1,2,3) - v(1,2)$
$\left| s \right|$	1	2	2	3
$w(\left| s \right|)$	1/3	1/6	1/6	1/3
$w(\left| s \right|)[v(s) - v(s\backslash 3)]$	$v(3)/3$	$[v(1,3) - v(1)]/6$	$[v(2,3) - v(2)]/6$	$[v(1,2,3) - v(1,2)]/3$
$\varphi_{3} (v)$	$v(3)/3 + [v(1,3) - v(1)]/6 + [v(2,3) - v(2)]/6 + [v(1,2,3) - v(1,2)]/3$			

#### Second layer: revenue allocation among participants of the same role

Once the Shapley values $\Phi (v) = (\varphi_{1} (v),\varphi_{2} (v),\varphi_{3} (v))$ for revenue allocation among the three roles in a road data asset are determined, it is necessary to determine the specific distribution of benefits to the participants under the same role based on the $\varphi_{i} (v)$ values of each role, to realize the distribution of benefits from the road data asset to each participant.

Assuming that there are $n$ participants in a road data asset revenue allocation, the number of participants with the roles of original data collectors, data processors, and data product producers is $n_{i}$($i = 1,2,3$), and it is clear that $n_{1} + n_{2} + n_{3} \ge n$. Denote $\varphi_{{_{j} }}^{i} (v)$ as the profit obtained by $j{\text{ th}}$ participant when distributing the profit $\varphi_{i} (v)$ of role $i$:

$$\varphi_{{_{j} }}^{i} (v) = \sum\limits_{{s \in s_{j}^{i} }} {w(\left| s \right|)[v(s) - v(s\backslash j)]\begin{array}{*{20}c} {} & {j = 1,2, \ldots ,n_{i} } \\ \end{array} }$$

$$w(\left| s \right|) = \frac{{(n_{i} - \left| s \right|)!(\left| s \right| - 1)!}}{{n_{i} !}}$$

4 $$\sum\limits_{j = 1}^{{n_{i} }} {\varphi_{j}^{i} } (v) = \varphi_{i} (v)\begin{array}{*{20}c} {} & {i = 1,2,3} \\ \end{array}$$

where $s_{{_{j} }}^{i}$ represents the set of all subsets of participants within role $i$ that includes participant $j$, $\left| s \right|$ is the number of elements in subset $s$, $w(\left| s \right|)$ is the weighting factor, $v(s)$ is the profit for subset $s$, and $v(s\backslash j)$ denotes the profit that can be obtained by excluding participant $j$ from subset $s$.

#### Synthesis of revenue allocation among participants

After calculating the revenue distribution for each participant within each role, it is necessary to synthesize the revenue distribution among participants under different roles, taking into account their contributions at different stages.

Let $N = \{ 1,2, \ldots ,n\}$ be the set of participants, and $N_{i} = \{ 1,2, \ldots ,n_{i} \}$($i = 1,2,3$) represents the set of participants for the roles of original data collectors, data processors, and data product producers, respectively. Clearly $N_{i} \subset N$, due to the different sizes and order of elements in sets $N$ and $N_{i}$, we define a function $f_{i} :N \to N_{i}$ that, for each element $x$($x = 1,2, \ldots n$) in set $N$, maps it to the corresponding element in set $N_{i}$, if there exists an element $j \in N_{i}$ such that $f_{i} (x) = j$, otherwise there is no corresponding element in set $N_{i}$. Therefore, the profit distribution for each participant $x$ in different roles $i$ can be represented as $\hat{\varphi }_{{_{x} }}^{i} (v)$, where:

5 $$\hat{\varphi }_{{_{x} }}^{i} (v) = \left\{ {\begin{array}{*{20}r} \hfill {\varphi_{{_{j} }}^{i} (v),\begin{array}{*{20}c} {} & {if} \\ \end{array} \begin{array}{*{20}c} {} & {f_{i} (x) = j} \\ \end{array} } \\ \hfill {0,\begin{array}{*{20}c} {} & {} & {if\begin{array}{*{20}c} {} & {f_{i} (x) \ne j} \\ \end{array} } \\ \end{array} } \\ \end{array} } \right.\begin{array}{*{20}c} {} & {i = 1,2,3} \\ \end{array}$$

To synthesize the profit values for each participant in different roles, we obtain the total profit distribution $\overline{\varphi }_{x} (v)$ for the participant in the road data asset, denoted as:

6 $$\overline{\varphi }_{x} (v) = \hat{\varphi }_{{_{x} }}^{1} (v) + \hat{\varphi }_{{_{x} }}^{2} (v) + \hat{\varphi }_{{_{x} }}^{3} (v)$$

### The modified Shapley value

**Figure 2:**
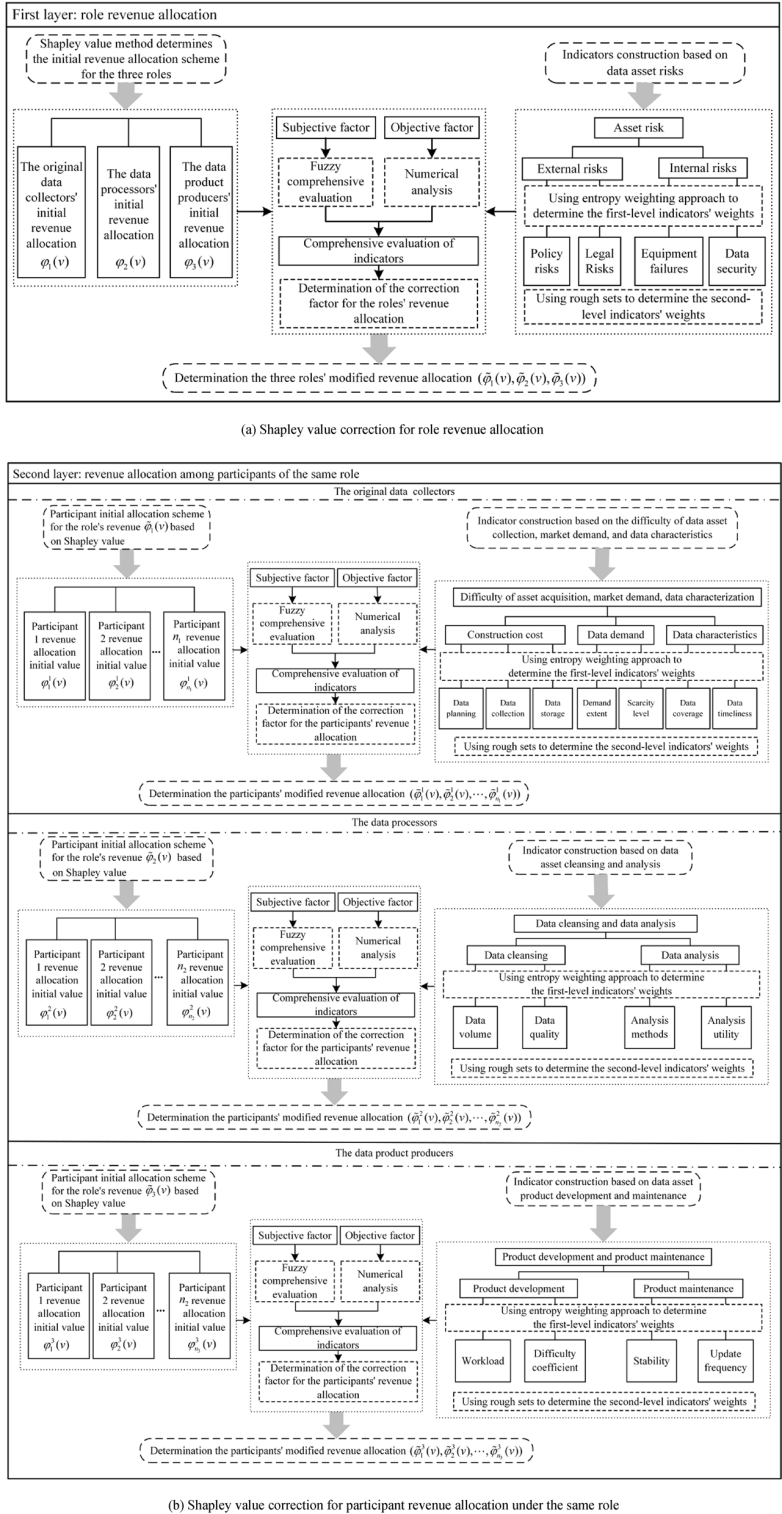

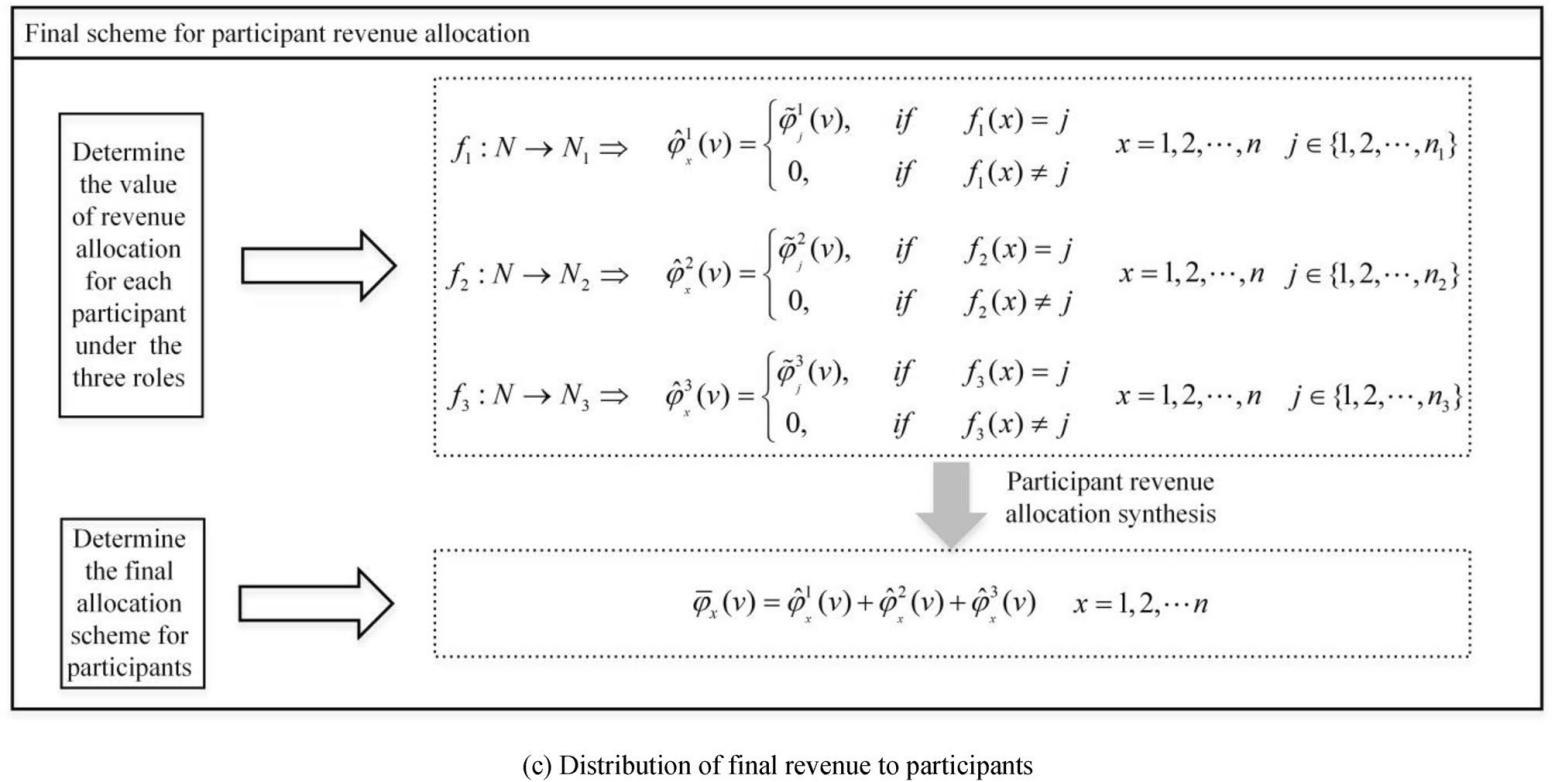
Architecture of the improved revenue allocation model for road data assets.

The traditional Shapley value method only determines revenue allocation based on marginal contributions, without considering differences among participants in terms of costs and risks. To achieve fair profit distribution of road data assets, it is essential to comprehensively evaluate the differences among roles and participants in terms of input costs, risk allocation, and other aspects. In this paper, based on the traditional Shapley value method, a revenue allocation evaluation indicator system for the road data asset, as depicted in Fig. 1, is established. This indicator-driven two-layer allocation correction scheme is used to modify the revenue allocation among different roles and participants. By doing so, a more equitable and reasonable revenue allocation model for the road data asset is developed. The architecture of the improved revenue allocation model for the road data asset is illustrated in Fig. 2.

#### Calculation of weights for evaluating revenue allocation of road data assets

##### Calculation of primary indicator weight

This study utilizes the entropy weighting method to calculate the weights of primary evaluation indicators in Fig. 1. It is assumed that $m$ expert will be invited to evaluate the importance of $I$ primary indicators and obtain a scoring matrix $S = (s_{ij} )_{m \times I}$, $i = 1,2, \ldots ,m$, $j = 1,2, \ldots ,I$, where $s_{ij}$ represents the rating provided by the $i$ expert for the $j$ indicator.

If $j$ denotes a profit-related indicator, normalization is performed according to Eq. (7):

7 $$\hat{s}_{ij} = \frac{{s_{ij} - \mathop {\min }\limits_{i} \{ s_{ij} \} }}{{\mathop {\max }\limits_{i} \{ s_{ij} \} - \mathop {\min }\limits_{i} \{ s_{ij} \} }}$$

If $j$ denotes a cost-related indicator, normalization is performed according to Eq. (8):

8 $$\hat{s}_{ij} = \frac{{\mathop {\max }\limits_{i} \{ s_{ij} \} - s_{ij} }}{{\mathop {\max }\limits_{i} \{ s_{ij} \} - \mathop {\min }\limits_{i} \{ s_{ij} \} }}$$

The weights $p_{ij}$ of the scores given by different experts to each indicator are calculated using the entropy weighting method, as shown in Eq. (9):

9 $$p_{ij} = \frac{{\hat{s}_{ij} }}{{\sum\limits_{i = 1}^{m} {\hat{s}_{ij} } }}$$

The information entropy value $e_{j}$ is calculated separately for each indicator $j$ according to $p_{ij}$:

10 $$e_{j} = - \frac{1}{\ln m}\sum\limits_{i = 1}^{m} {p_{ij} \ln p_{ij} }$$

To ensure that the entropy value $e_{j}$ holds numerical significance, we set $\ln p_{ij} = 0$ when $p_{ij} = 0$.

The entropy weight $\omega_{j}$ for each indicator is then calculated based on the entropy value $e_{j}$, as follows:

11 $$\omega_{j} = \frac{{1 - e_{j} }}{{\sum\limits_{j = 1}^{I} {(1 - e_{j} )} }}$$

##### Calculation of secondary indicator weight

For the secondary evaluation indicators in Fig. 1, the rough set theory is employed in this study to calculate their indicator weights. It is assumed that $m$ experts are invited to assess the importance of $I_{j}$ secondary indicators under the $j$($j = 1,2, \ldots ,I$) primary indicator, leading to the construction of an evaluation information system $S_{j} = (U,A_{j} ,V_{j} ,f)$, where: the universe of discourse $U = \{ 1,2, \ldots ,m\}$, a non-empty finite attribute set $A_{j} = \{ a_{1} ,a_{2} , \ldots ,a_{{I_{j} }} \}$, and the attribute value domain $V_{j}$ are obtained through expert assessment using a percentage-based scoring system. Moreover, $f$ represents the relationship set between $U$ and $A_{j}$, also referred to as the information function set.

###### Definition 1

Let $R$ be an equivalence relation on $U$, denoted as:

12 $$ind(R) = \{ (x,y) \in U \times U|\forall a \in A_{j} ,f(x,a) = f(y,a)\}$$

$U/ind(R)$ is referred to as the partition of $U$, and each element $a$ is called an equivalence class.

In an information system $S_{j}$, different attributes have varying effects, and some attributes may even be redundant. Therefore, it is necessary to eliminate irrelevant or unimportant knowledge from the information system while maintaining its classification ability. This process is known as knowledge reduction. Knowledge reduction is divided into attribute reduction and attribute value reduction. However, since attribute value reduction is relatively straightforward, knowledge reduction generally refers to attribute reduction in most cases.

###### Definition 2

If $ind(R) = ind(R - \{ r\} )$, it is referred to $r$ as reducible knowledge in the information system $R$. If $P = R - \{ r\}$ is independent, then $P$ is a knowledge reduction in $R$.

In the information system $S_{j}$, the set of secondary indicators for the primary indicator $j$ is denoted as $A_{j} = \{ a_{1} ,a_{2} , \ldots ,a_{{I_{j} }} \}$. Assume that there are $l_{j}$ sets of $A_{j}$ divisions over $U$, represented as $U/ind(A) = \{ X_{1} ,X_{2} , \ldots ,X_{{l_{j} }} \}$. The information quantity of $A_{j}$ is calculated as:

13 $$I(A_{j} ) = \sum\limits_{i = 1}^{{l_{j} }} {\frac{{\left| {X_{i} } \right|}}{\left| U \right|}\left[ {1 - \frac{{\left| {X_{i} } \right|}}{\left| U \right|}} \right]} = 1 - \frac{1}{{\left| U \right|^{2} }}\sum\limits_{i = 1}^{{l_{j} }} {\left| {X_{i} } \right|^{2} }$$

where $\left| U \right|$ represents the number of elements in the universe of discourse $U$, and $\left| {X_{i} } \right|$ denotes the number of elements in the $i{\text{ th}}$ set.

In the information system $S_{j}$, for the knowledge reduction $ind(A_{j} - \{ a\} )$ of $\forall a \in A_{j}$, let there exist $l_{a}$ sets of the partition of $U$ after reduction, denoted as $U/ind(A_{j} - \{ a\} ) = \{ X_{1} ,X_{2} , \ldots ,X_{{l_{a} }} \}$. The information quantity of $A_{j} - \{ a\}$ is given by:

14 $$I(A_{j} - \{ a\} ) = \sum\limits_{i = 1}^{{l_{a} }} {\frac{{\left| {X_{i} } \right|}}{\left| U \right|}\left[ {1 - \frac{{\left| {X_{i} } \right|}}{\left| U \right|}} \right]} = 1 - \frac{1}{{\left| U \right|^{2} }}\sum\limits_{i = 1}^{{l_{a} }} {\left| {X_{i} } \right|^{2} }$$

Therefore, the importance of $a$ in $A_{j}$ can be expressed as:

15 $$Sig_{{A_{j} }} (a) = I(A_{j} ) - I(A_{j} - \{ a\} )$$

The weights of secondary indicators $A_{j} = \{ a_{1} ,a_{2} , \ldots ,a_{{I_{j} }} \}$ under the primary indicator $j$ can be calculated based on their importance using the equation:

16 $$\omega_{{A_{j} }} (a) = \frac{{Sig_{{A_{j} }} (a)}}{{\sum\limits_{a = 1}^{{I_{j} }} {Sig_{{A_{j} }} (a)} }}$$

By incorporating the entropy weight $\omega_{j}$ of the primary indicator $j$, the final weights of the secondary indicators $A_{j} = \{ a_{1} ,a_{2} , \ldots ,a_{{I_{j} }} \}$ can be determined as:

17 $$\tilde{\omega }_{{A_{j} }} (a) = \omega_{j} \times \omega_{{A_{j} }} (a),\;\;\;\;\;a = 1,2, \cdots I_{j} ,j = 1,2, \ldots ,I$$

#### Evaluation of revenue allocation indicators for road data assets

Once the weights of the revenue allocation evaluation indicators for road data assets are determined, it is necessary to numerically evaluate different schemes under the relevant indicators. As some indicators involve subjective measures and others are objective numerical metrics, different methods are required to quantify both subjective and objective factors for an effective assessment of revenue allocation indicators for road data assets.

Defining a scheme as a collective term for subjects involved in revenue allocation across different layers, the scheme represents roles at the first layer and participants within each role at the second layer. Assuming that there are $D$ schemes involved in the distribution of a road data asset, scheme $d$($d = 1,2, \ldots ,D$), requires a comprehensive evaluation of all secondary indicators under $I$ primary indicators. Let there be $I_{j}$ secondary indicators under the $j{\text{ th}}$($j = 1,2, \ldots ,I$) primary indicator, and the set of indicators is $A_{j} = \{ a_{1} ,a_{2} , \ldots ,a_{{I_{j} }} \}$, of which there are $\dot{I}_{j}$ subjective indicators and $\ddot{I}_{j}$ objective indicators, and $\dot{I}_{j} + \ddot{I}_{j} = I_{j}$, let the set of subjective secondary indicators under the $j{\text{ th}}$ primary indicator be $A^{\prime}_{j} = \{ a^{\prime}_{1} ,a^{\prime}_{2} , \ldots ,a^{\prime}_{{\dot{I}_{j} }} \}$, $a^{\prime}_{i} \in A_{j} ,i = 1,2, \ldots ,\dot{I}_{j}$, and the set of objective secondary indicators be $A^{\prime\prime}_{j} = \{ a^{\prime\prime}_{1} ,a^{\prime\prime}_{2} , \ldots ,a^{\prime\prime}_{{\ddot{I}_{j} }} \}$, $a^{\prime\prime}_{i} \in A_{j} ,i = 1,2, \ldots ,\ddot{I}_{j}$, and $A^{\prime}_{j} \cup A^{\prime\prime}_{j} = A_{j}$, $A^{\prime}_{j} \cap A^{\prime\prime}_{j} = \emptyset$.

##### Subjective evaluation

Suppose $m$ experts are invited to assess scheme $d$ based on the subjective indicator set $A^{\prime}_{j} = \{ a^{\prime}_{1} ,a^{\prime}_{2} , \ldots ,a^{\prime}_{{\dot{I}_{j} }} \}$ for indicator $j$. Based on the comment set $V =${*low*,* moderately low*, *moderate*, *moderately high*,* high*}, a fuzzy evaluation is conducted to obtain the fuzzy relationship matrix:

18 $$R_{j} (d) = \left[ {\begin{array}{*{20}c} {r_{{a^{\prime}_{1} }}^{1} (d)} & {r_{{a^{\prime}_{1} }}^{2} (d)} & {r_{{a^{\prime}_{1} }}^{3} (d)} & {r_{{a^{\prime}_{1} }}^{4} (d)} & {r_{{a^{\prime}_{1} }}^{5} (d)} \\ {r_{{a^{\prime}_{2} }}^{1} (d)} & {r_{{a^{\prime}_{2} }}^{2} (d)} & {r_{{a^{\prime}_{2} }}^{3} (d)} & {r_{{a^{\prime}_{2} }}^{4} (d)} & {r_{{a^{\prime}_{2} }}^{5} (d)} \\ \vdots & \vdots & \vdots & \vdots & \vdots \\ {r_{{a^{\prime}_{{\dot{I}_{j} }} }}^{1} (d)} & {r_{{a^{\prime}_{{\dot{I}_{j} }} }}^{2} (d)} & {r_{{a^{\prime}_{{\dot{I}_{j} }} }}^{3} (d)} & {r_{{a^{\prime}_{{\dot{I}_{j} }} }}^{4} (d)} & {r_{{a^{\prime}_{{\dot{I}_{j} }} }}^{5} (d)} \\ \end{array} } \right]$$

Among them, $r_{{a^{\prime}_{i} }}^{1} (d)$, $r_{{a^{\prime}_{i} }}^{2} (d)$, $r_{{a^{\prime}_{i} }}^{3} (d)$, $r_{{a^{\prime}_{i} }}^{4} (d)$, and $r_{{a^{\prime}_{i} }}^{5} (d)$ respectively represent the frequency distribution of indicator $a^{\prime}_{i}$($i = 1,2, \ldots ,\dot{I}_{j}$) under the five comments of low, moderately low, moderate, moderately high, and high.

Based on the indicator weights calculated according to Eq. (17), the subjective indicator weight vector for Indicator $A^{\prime}_{j}$ is denoted as $\tilde{\omega }_{{A^{\prime}_{j} }} = [\tilde{\omega }_{{A^{\prime}_{j} }} (a^{\prime}_{1} ),\tilde{\omega }_{{A^{\prime}_{j} }} (a^{\prime}_{2} ), \ldots ,\tilde{\omega }_{{A^{\prime}_{j} }} (a^{\prime}_{{\dot{I}_{j} }} )]$. Using this weight vector, the fuzzy evaluation vector is obtained as:

19 $$T_{j} (d) = \tilde{\omega }_{{A^{\prime}_{j} }} \times R_{j} (d)$$

where $T_{j} (d)$ is referred to as the fuzzy evaluation vector.

Using the membership degree of the comment set $V =${*low, moderately low, moderate, moderately high, high*}, the membership degree vector $\overline{V} = [0.1,0.3,0.5,0.7,0.9]$ can be determined. From this, the evaluation value $L^{\prime}_{j} (d)$ of the subjective component for indicator $j$ can be calculated as:

20 $$L^{\prime}_{j} (d) = T_{j} (d) \times \overline{V}^{T}$$

The subjective evaluation values for $I$ primary indicators are synthesized as:

21 $$L^{\prime}(d) = \sum\limits_{j = 1}^{I} {L^{\prime}_{j} (d)}$$

where $L^{\prime}(d)$ is termed as the subjective evaluation value of scheme $d$.

#### Objective evaluation

In the set of objective indicators $A^{\prime\prime}_{j} = \{ a^{\prime\prime}_{1} ,a^{\prime\prime}_{2} , \ldots ,a^{\prime\prime}_{{\ddot{I}_{j} }} \}$, the numerical value for each indicator of Scheme $d$ is represented by a vector, denoted as $f_{d} (A^{\prime\prime}_{j} ) = [f_{d} (a^{\prime\prime}_{1} ),f_{d} (a^{\prime\prime}_{2} ), \ldots ,f_{d} (a^{\prime\prime}_{{\ddot{I}_{j} }} )]$. For the indicator $a^{\prime\prime}_{i}$($i = 1,2, \ldots ,\ddot{I}_{j}$), if it is a revenue indicator, it is normalized on the scheme $D$ according to Eq. (22), and if it is a cost indicator, the values are normalized using Eq. (23). This normalization process yields the normalized value vector, denoted as $\tilde{f}_{d} (A^{\prime\prime}_{j} ) = [\tilde{f}_{d} (a^{\prime\prime}_{1} ),\tilde{f}_{d} (a^{\prime\prime}_{2} ), \ldots ,\tilde{f}_{d} (a^{\prime\prime}_{{\ddot{I}_{j} }} )]$, where it is evident that $\sum\limits_{d = 1}^{D} {\tilde{f}_{d} (a^{\prime\prime}_{i} } ) = 1$.

22 $$\tilde{f}_{d} (a^{\prime\prime}_{i} ) = \frac{{f_{d} (a^{\prime\prime}_{i} )}}{{\sum\limits_{d = 1}^{D} {f_{d} (a^{\prime\prime}_{i} } )}}$$

23 $$\tilde{f}_{d} (a^{\prime\prime}_{i} ) = 1 - \frac{{f_{d} (a^{\prime\prime}_{i} )}}{{\sum\limits_{d = 1}^{D} {f_{d} (a^{\prime\prime}_{i} } )}}$$

According to the weights of the indicators calculated in Eq. (17), the weight vector $\tilde{\omega }_{{A^{\prime\prime}_{j} }} = [\tilde{\omega }_{{A^{\prime\prime}_{j} }} (a^{\prime\prime}_{1} ),\tilde{\omega }_{{A^{\prime\prime}_{j} }} (a^{\prime\prime}_{2} ), \ldots ,\tilde{\omega }_{{A^{\prime\prime}_{j} }} (a^{\prime\prime}_{{\ddot{I}_{j} }} )]$ of the objective indicator $A^{\prime\prime}_{j}$ can be obtained, and based on the vector of normalized values $\tilde{f}_{d} (A^{\prime\prime}_{j} )$, the evaluation value of the objective part of the indicator $j$ is calculated as $L^{\prime\prime}_{j} (d)$:

24 $$L^{\prime\prime}_{j} (d) = \tilde{\omega }_{{A^{\prime\prime}_{j} }} \times \tilde{f}_{d} (A^{\prime\prime}_{j} )^{T}$$

Synthesize the assessed value of the objective component of the $I$ primary indicators indicator:

25 $$L^{\prime\prime}(d) = \sum\limits_{j = 1}^{I} {L^{\prime\prime}_{j} (d)}$$

where $L^{\prime\prime}(d)$ is called the objective evaluation value of scheme $d$.

#### Integration of objective and subjective evaluations

Combine the subjective and objective evaluation values for scheme $d$ to obtain the composite evaluation value.

26 $$L(d) = \alpha L^{\prime}(d) + (1 - \alpha )L^{\prime\prime}(d),d = 1,2, \ldots ,D$$

where $L(d)$ is the composite evaluated value of scheme $d$ and $\alpha$($0 \le \alpha \le 1$) is the weighting factor, allowing for the adjustment of the importance of subjective and objective evaluation values in the composite evaluated value.

Normalize the composite evaluated value $L(d)$ of scheme $d$:

27 $$\tilde{L}(d) = \frac{L(d)}{{\sum\limits_{d = 1}^{D} {L(d)} }},d = 1,2, \ldots ,D$$

#### The modification of road data asset revenue allocation

##### Role revenue allocation modification

Based on Eq. (3), the initial allocations for the roles $R = \{ 1,2,3\}$ of the original data collectors, data processors, and data product producers can be computed, denoted as $\Phi (v) = (\varphi_{1} (v),\varphi_{2} (v),\varphi_{3} (v))$. Additionally, it is known that $\varphi_{1} (v) + \varphi_{2} (v) + \varphi_{3} (v) = v(R)$, where $v(R)$ represents the maximum revenue for the road data asset.

As illustrated in Fig. 2a, based on the model in Section "Evaluation of revenue allocation indicators for road data assets", the comprehensive evaluation values $\tilde{L}_{R} (1)$, $\tilde{L}_{R} (2)$, and $\tilde{L}_{R} (3)$ for the roles of the original data collectors, data processors, and data product producers can be calculated.

Next, compute the role revenue allocation modification factor:

28 $$\Delta \theta_{i} = \tilde{L}_{R} (i) - \frac{1}{3},i = 1,2,3$$

The modified value of the role's revenue allocation is:

29 $$\tilde{\varphi }_{i} (v) = \varphi_{i} (v) + \Delta \theta_{i} \times v(R),i = 1,2,3$$

### Participant revenue allocation modification within the same role

Suppose there are $n$ participants involved in the distribution of road data asset profits, and the number of participants in the roles of data collectors, data processors, and data product producers is denoted as $n_{i}$($i = 1,2,3$). According to Eq. (4), we determine the initial distribution scheme $\Phi^{i} (v) = (\varphi_{{_{1} }}^{i} (v),\varphi_{{_{2} }}^{i} (v), \ldots ,\varphi_{{_{{n_{i} }} }}^{i} (v))$ of participants within the role $i$ based on the role revenue allocation modified value $\tilde{\varphi }_{i} (v)$, where $\sum\limits_{j = 1}^{{n_{i} }} {\varphi_{j}^{i} (v)} = \tilde{\varphi }_{i} (v),i = 1,2,3$.

As shown in Fig. 2b, applying the model in Section "Evaluation of revenue allocation indicators for road data assets", we can calculate the comprehensive evaluation value $[\tilde{L}^{1} (1),\tilde{L}^{1} (2), \ldots ,\tilde{L}^{1} (n_{1} )]$ for participants within the data collectors.

To modify the revenue allocation for participants within the data collectors, we compute the participant modification factor as follows:

30 $$\Delta \theta_{j}^{1} = \tilde{L}^{1} (j) - \frac{1}{{n_{1} }},j = 1,2, \ldots ,n_{1}$$

The modified values for participant revenue allocation within the data collectors are then given by:

31 $$\tilde{\varphi }_{j}^{1} (v) = \varphi_{j}^{1} (v) + \Delta \theta_{j}^{1} \times \tilde{\varphi }_{1} (v)$$

Similarly, using the model in Section "Evaluation of revenue allocation indicators for road data assets", we can calculate the comprehensive evaluation value $[\tilde{L}^{2} (1),\tilde{L}^{2} (2), \ldots ,\tilde{L}^{2} (n_{2} )]$ for participants within the data processors.

For the data processors, the participant revenue allocation modification factor is calculated as follows:

32 $$\Delta \theta_{j}^{2} = \tilde{L}^{2} (j) - \frac{1}{{n_{2} }},j = 1,2, \ldots ,n_{2}$$

The modified values for participant revenue allocation within the data processors are then obtained as:

33 $$\tilde{\varphi }_{j}^{2} (v) = \varphi_{j}^{2} (v) + \Delta \theta_{j}^{2} \times \tilde{\varphi }_{2} (v)$$

Likewise, considering the model in Section "Evaluation of revenue allocation indicators for road data assets", we can compute the comprehensive evaluation value $[\tilde{L}^{3} (1),\tilde{L}^{3} (2), \ldots ,\tilde{L}^{3} (n_{3} )]$ for participants within the data product producers.

To modify the revenue allocation for participants within the data product producers, we calculate the participant revenue allocation modification factor as follows:

34 $$\Delta \theta_{j}^{3} = \tilde{L}^{3} (j) - \frac{1}{{n_{3} }},j = 1,2, \ldots ,n_{3}$$

Finally, the modified values for participant revenue allocation within the data product producers are given by:

35 $$\tilde{\varphi }_{j}^{3} (v) = \varphi_{j}^{3} (v) + \Delta \theta_{j}^{3} \times \tilde{\varphi }_{3} (v)$$

#### Final revenue allocation scheme for participants

As depicted in Fig. 2(c), using Eq. (5), we determine the revenue allocation modified values for the $n$ participants across the three roles:

36 $$\hat{\varphi }_{{_{x} }}^{i} (v) = \left\{ {\begin{array}{*{20}r} \hfill {\tilde{\varphi }_{{_{j} }}^{i} (v),\begin{array}{*{20}c} {} & {if} \\ \end{array} \begin{array}{*{20}c} {} & {f_{i} (x) = j} \\ \end{array} } \\ \hfill {0,\begin{array}{*{20}c} {} & {} & {if\begin{array}{*{20}c} {} & {f_{i} (x) \ne j} \\ \end{array} } \\ \end{array} } \\ \end{array} } \right.\begin{array}{*{20}c} {} & {x = 1,2, \ldots ,n;\begin{array}{*{20}c} {} \\ \end{array} i = 1,2,3} \\ \end{array}$$

By synthesizing the profit values for each participant across the different roles, we obtain the final revenue allocation values for each participant involved in the road data asset:

37 $$\overline{\varphi }_{x} (v) = \hat{\varphi }_{{_{x} }}^{1} (v) + \hat{\varphi }_{{_{x} }}^{2} (v) + \hat{\varphi }_{{_{x} }}^{3} (v)\begin{array}{*{20}c} {} & {x = 1,2, \ldots ,n} \\ \end{array}$$

## Case study

Assuming that the sale of a road data asset obtains total proceeds of 960,000 RMB, the revenue need to be allocated to the five enterprises $N = \{ 1,2,3,4,5\}$ involved in data collection, processing and production. According to the process of realizing the value of road data, enterprises can be divided into three types of roles $R = \{ 1,2,3\}$: the original data collectors, the data processors and the data product producers, and the set of participating enterprises under the three types of roles are $N_{1} = \{ 1,2\}$, $N_{2} = \{ 2,3,4\}$, and $N_{3} = \{ 4,5\}$, respectively. Based on our investigation, we found that selling the original data directly can generate revenue of 300,000 RMB while processing the original data and selling it can bring in revenue of 420,000 RMB. Developing the original data into data products and selling them can yield revenue of 660,000 RMB. Without the original data, neither the data processors nor the data product producers can generate any revenue, regardless of whether they operate individually or in cooperation.

The income values and indicator values for the participating enterprises in each role are reasonably assumed, as shown in Tables 4, 5, 6 and 7.

**Table 4:** Revenue situation of participating enterprise combinations under each role (unit: ten thousand RMB).

Combination of the enterprises	The original data collectors (1)			The data processors (2)							The data product producers (3)		
$s$	$\{ 1\}$	$\{ 2\}$	$\{ {1,}2\}$	$\{ 2\}$	$\{ 3\}$	$\{ 4\}$	$\{ 2,3\}$	$\{ 2,4\}$	$\{ 3,4\}$	$\{ {2,}3,4\}$	$\{ 4\}$	$\{ 5\}$	$\{ {4,}5\}$
$v(s)$	10	16	30	1.2	3	2.4	6	4.8	6.6	12	8	12	33

**Table 5:** Indicator values for participating enterprises under the original data collectors.

Participating enterprises			Enterprise 1	Enterprise 2
Construction cost	Data planning (unit: ten thousand RMB)		3	2.5
	Data collection (unit: ten thousand RMB)		45	30
	Data storage (unit: ten thousand RMB)		6	10
Data demand	Demand level		Qualitative evaluation	
	Scarcity level			
Data characteristics	Data coverage	Time coverage (unit: year)	2	3
		Space coverage (unit: road section)	A road section in Gansu Province, China	
	Data timeliness (unit: minute)		30	10

**Table 6:** Indicator values for participating enterprises under the data processors.

Participating enterprises			Enterprise 2	Enterprise 3	Enterprise 4
Data cleansing	Data volume (unit: record)		25,000	45,000	30,000
	Data quality	Completeness (unit: %, the original data is 70%)	95	98	99%
		Validity (unit: %, the original data is 82%)	100	97	98%
		Consistency (unit: %, the original data is 86%)	90	93	95%
Data analysis	Analysis methods		Qualitative evaluation		
	Analysis utility

**Table 7:** Indicator values for participating enterprises under the data product producers.

Participating enterprises		Enterprise 4	Enterprise 5
Product development	Workload (unit: line)	15,000	20,000
	Difficulty coefficient	0.8	0.95
Product maintenance	Stability (unit: days/month)	29	27
	Update frequency (unit: month)	3	2

### Role revenue allocation

With reference to the revenue data in Table 3, the initial revenue allocation for the original data collectors, the data processors, and the data product producers is calculated using the traditional Shapley value method, as presented in Table 8.

**Table 8:** Initial revenue allocation for the three roles.

Role $i$	The original data collectors (1)				The data processors (2)				The data product producers (3)			
*s*	{1}	{1,2}	{1,3}	{1,2,3}	{2}	{1,2}	{2,3}	{1,2,3}	{3}	{1,3}	{2,3}	{1,2,3}
$v(s)$	30	42	66	96	0	42	0	96	0	66	0	96
$v(s\backslash i)$	0	0	0	0	0	30	0	66	0	30	0	42
$v(s) - v(s\backslash i)$	30	42	66	96	0	12	0	30	0	36	0	54
$\left| s \right|$	1	2	2	3	1	2	2	3	1	2	2	3
$w(\left| s \right|)$	$1/3$	$1/6$	$1/6$	$1/3$	$1/3$	$1/6$	$1/6$	$1/3$	$1/3$	$1/6$	$1/6$	$1/3$
$w(\left| s \right|)[v(s) - v(s\backslash i)]$	10	7	11	32	0	2	0	10	0	6	0	18
$\varphi_{i} (v)$	60				12				24			

Determine the weights of the evaluation indexes for the role revenue allocation. Ten experts in the field of road data assets were asked to evaluate the importance of two primary indicators, external risk, and internal risk, using a 1–9 scale. The weights of these indicators were then determined using the entropy weight method. The scoring results provided by the experts are presented in Table 9.

**Table 9:** Scoring results of the role's primary indicators.

$A$	External risks $A_{1}$	Internal risks $A_{2}$
Expert 1	6	7
Expert 2	7	8
Expert 3	5	5
Expert 4	7	5
Expert 5	6	9
Expert 6	8	7
Expert 7	5	9
Expert 8	4	7
Expert 9	9	7
Expert 10	7	7

According to Eqs. (7) and (9), the scoring results were normalized and the weights $p_{ij}$ were calculated as shown in Table 10.

**Table 10:** Scoring weights for the role's primary indicators.

$A$	External risks $A_{1}$	Internal risks $A_{2}$
Expert 1	0.083	0.095
Expert 2	0.125	0.143
Expert 3	0.042	0.000
Expert 4	0.125	0.000
Expert 5	0.083	0.191
Expert 6	0.167	0.095
Expert 7	0.042	0.191
Expert 8	0.000	0.095
Expert 9	0.208	0.095
Expert 10	0.125	0.095

The information entropy values and entropy weights of the indicators were calculated according to Eqs. (10) and (11), as shown in Table 11. The weights for the primary evaluation indicators of the roles, denoted as $\omega_{1} = 0.444$ and $\omega_{2} = 0.556$, were obtained.

**Table 11:** Process of calculating entropy weights for the role's primary indicators.

$j$	External risks $A_{1}$	Internal risks $A_{2}$
$e_{j}$	0.905	0.881
$1 - e_{j}$	0.095	0.119
$\omega_{j}$	0.444	0.556

The secondary evaluation indicators for the roles were scored on a percentage scale, with higher scores indicating greater importance of the indicators. The scoring results are presented in Table 12.

**Table 12:** Scoring results of the roles secondary indicators.

$A$	External risks $A_{1}$		Internal risks $A_{2}$	
	Policy risks	Legal risks	Equipment failures	Data security
Expert 1	85	80	80	95
Expert 2	80	80	76	96
Expert 3	70	85	70	90
Expert 4	68	76	65	87
Expert 5	75	90	75	95
Expert 6	84	88	82	93
Expert 7	73	89	90	90
Expert 8	83	86	68	89
Expert 9	90	95	90	99
Expert 10	82	85	81	97

To facilitate further analysis and capture more common features in the sample data, it is necessary to abstract the indicator scores into higher-level data. Considering the simplification of the model, an unsupervised distance-based method was employed in this study to classify the expert scoring results into three categories, as shown in Table 13. In future research, more scientifically designed and applicable classification methods can be developed based on the characteristics of the scoring data to enhance effectiveness and reliability.

**Table 13:** Classification results of secondary indicator scores for roles.

$A$	External risks $A_{1}$		Internal risks $A_{2}$	
	Policy risks	Legal risks	Equipment failures	Data security
Expert 1	3	1	2	2
Expert 2	2	1	2	3
Expert 3	1	2	1	1
Expert 4	1	1	1	1
Expert 5	1	2	2	2
Expert 6	2	2	2	2
Expert 7	1	2	3	1
Expert 8	2	2	1	1
Expert 9	3	3	3	3
Expert 10	2	2	2	3

The weights of the secondary indicators under external risks and internal risks were calculated according to Eqs. (13)–(16), as shown in Table 14. It is worth noting that the elements within the sets in Table 14 correspond to the indices of the scoring experts in Table 13.

**Table 14:** Process of calculating weights for the role's secondary indicators.

$A$	External risks $A_{1}$		Internal risks $A_{2}$	
$U/ind(A{)}$	${{\{ \{ 1\} ,\{ 2\} ,\{ 3,5,7\} ,\{ 4\} ,\{ 6,8,10\} ,\{ 9\} \} }}$		${{\{ \{ 1,5,6\} ,\{ 2,10\} ,\{ 3,4,8\} ,\{ 7\} ,\{ 9\} \} }}$	
$I(A)$	0.780		0.760	
$a$	Policy risks $a_{1}$	Legal risks $a_{2}$	Equipment failures $a_{1}$	Data security $a_{2}$
$U/ind(A - \{ a\} )$	$$\begin{gathered} \{ \{ 1,2,4\} ,\{ 9\} , \{ 3,5,6,7,8,10\} \} \end{gathered}$$	$$\begin{gathered} \{ \{ 1,9\} ,\{ 2,6,8,10\} , \{ 3,4,5,7\} \} \end{gathered}$$	$$\begin{gathered} \{ \{ 1,5,6\} ,\{ 2,9,10\} , \{ 3,4,7,8\} \} \end{gathered}$$	$$\begin{gathered} \{ \{ 1,2,5,6,10\} , \{ 3,4,8\} ,\{ 7,9\} \} \end{gathered}$$
$I(A - \{ a\} )$	0.540	0.640	0.660	0.620
$Sig_{A} (a)$	0.240	0.140	0.100	0.140
$\omega_{A} (a)$	0.630	0.370	0.420	0.580

Using Eq. (17), the final weights for the secondary indicators under external risks and internal risks are $\tilde{\omega }_{{A_{1} }} = [0.280,0.164]$ and $\tilde{\omega }_{{A_{2} }} = [0.234,0.322]$, respectively.

The evaluation of the secondary indicators under external risks and internal risks for each role is subjective. The evaluation process for the indicators of each role is shown in Table 15.

**Table 15:** The risk indicator evaluation process for each role.

$d$	$j$	$R_{j} (d)$	$L^{\prime}_{j} (d)$	$L^{\prime}(d)$
The original data collectors	External risks	$$\left[ {\begin{array}{*{20}c} 0 & {0.2} & {0.3} & {0.2} & {0.3} \\ {0.1} & {0.2} & {0.5} & {0.2} & 0 \\ \end{array} } \right]$$	0.249	0.586
	Internal risks	$$\left[ {\begin{array}{*{20}c} 0 & {0.2} & {0.4} & {0.3} & {0.1} \\ 0 & {0.1} & {0.3} & {0.4} & {0.2} \\ \end{array} } \right]$$	0.337	
The data processors	External risks	$$\left[ {\begin{array}{*{20}c} {0.3} & {0.2} & {0.3} & {0.1} & {0.1} \\ {0.1} & {0.1} & {0.3} & {0.3} & {0.2} \\ \end{array} } \right]$$	0.207	0.449
	Internal risks	$$\left[ {\begin{array}{*{20}c} {0.3} & {0.2} & {0.3} & {0.1} & {0.1} \\ {0.2} & {0.2} & {0.3} & {0.2} & {0.1} \\ \end{array} } \right]$$	0.242	
The data product producers	External risks	$$\left[ {\begin{array}{*{20}c} {0.1} & {0.2} & {0.5} & {0.2} & 0 \\ {0.2} & {0.3} & {0.3} & {0.1} & {0.1} \\ \end{array} } \right]$$	0.197	0.488
	Internal risks	$$\left[ {\begin{array}{*{20}c} {0.1} & {0.1} & {0.3} & {0.3} & {0.2} \\ {0.2} & {0.2} & {0.2} & {0.3} & {0.1} \\ \end{array} } \right]$$	0.291	

Since the evaluation indicators for the original data collectors, data processors, and data product producers are all subjective indicators, according to Eqs. (26) and (27), in this case, we take $\alpha = 1$ and calculate the normalized comprehensive correction values for the three categories of roles as $\tilde{L}_{R} (1) = 0.38{5}$, $\tilde{L}_{R} (2) = 0.{295}$, and $\tilde{L}_{R} (3) = 0.32{0}$. Furthermore, we can calculate the modified revenue allocation values for the three categories of roles as $\tilde{\varphi }_{1} (v) = 64.{960}$, $\tilde{\varphi }_{2} (v) = 8.{320}$, and $\tilde{\varphi }_{3} (v) = 22.72{0}$.

### Revenue allocation among participants of the same role

#### Revenue allocation among participants of the original data collectors

According to Eq. (4), the modified revenue allocation from the original data collectors to Enterprise 1 and Enterprise 2 is calculated as $\varphi_{1}^{1} (v) = 29.{48}$ and $\varphi_{2}^{1} (v) = 35.{48}$.

Using the entropy weight method to calculate the weights of the primary indicators that influence the revenue allocation for the participants under the original data collectors, similar to Sect. 4.1, the weights assigned by experts are shown in Table 16.

**Table 16:** The weighting of primary indicator scores for the original data collectors.

$A$	Construction cost $A_{3}$	Data demand $A_{4}$	Data characteristics $A_{5}$
Expert 1	0.105	0	0.215
Expert 2	0.053	0.118	0
Expert 3	0.211	0.176	0.143
Expert 4	0.105	0.118	0.071
Expert 5	0.053	0	0.071
Expert 6	0.105	0.118	0
Expert 7	0	0.118	0.071
Expert 8	0.158	0.118	0.215
Expert 9	0.105	0.176	0.071
Expert 10	0.105	0.058	0.143

The information entropy values and entropy weights of the primary indicators for the original data collectors are calculated, resulting in indicator weights of $\omega_{3} = 0.238$, $\omega_{4} = 0.337$, and $\omega_{5} = 0.425$, as shown in Table 17.

**Table 17:** Process of calculating entropy weights for the primary indicators of the original data collectors.

$j$	Construction cost $A_{3}$	Data demand $A_{4}$	Data characteristics $A_{5}$
$e_{j}$	0.919	0.885	0.855
$1 - e_{j}$	0.081	0.115	0.145
$\omega_{j}$	0.238	0.337	0.425

The weights of the secondary indicators for the original data collectors are determined, and the classification results of the expert scores are shown in Table 18.

**Table 18:** Classification results of secondary indicator scores for the original data collectors.

$A$	Construction cost $A_{3}$			Data demand $A_{4}$		Data characteristics $A_{5}$	
	Data planning	Data collection	Data storage	Demand level	Scarcity level	Data coverage	Data timeliness
Expert 1	1	3	3	1	2	1	1
Expert 2	2	2	3	2	2	1	2
Expert 3	1	3	3	3	1	3	2
Expert 4	2	2	1	2	2	2	3
Expert 5	2	1	2	1	1	1	1
Expert 6	3	1	1	1	1	2	3
Expert 7	3	2	1	3	3	1	2
Expert 8	3	1	1	1	1	1	1
Expert 9	2	2	3	3	3	3	3
Expert 10	1	1	3	1	1	1	1

The weights of the secondary indicators that influence the participants under the original data collectors are calculated according to Eqs. (13)–(16), and the specific process is displayed in Table 19. It is worth noting that the elements within the sets in Table 19 correspond to the indices of the scoring experts in Table 18.

**Table 19:** Process of calculating weights for the secondary indicators of the original data collectors.

$A$	Construction cost $A_{3}$			Data demand $A_{4}$		Data characteristics $A_{5}$	
$U/ind(A{)}$	$$\begin{gathered} \{ \{ 1,3\} ,\{ 2,9\} ,\{ 4\} , \{ 5\} ,\{ 6,8\} ,\{ 7\} ,\{ 10\} \} \end{gathered}$$			$$\begin{gathered} \{ \{ 1\} ,\{ 2,4\} ,\{ 3\} ,\{ 7\} , \{ 5,6,8,10\} ,\{ 9\} \} \end{gathered}$$		$$\begin{gathered} \{ \{ 1,5,8,10\} ,\{ 2,7\} , \{ 3\} ,\{ 4,6\} ,\{ 9\} \} \end{gathered}$$	
$I(A)$	0.840			0.760		0.740	
$a$	Data planning $a_{1}$	Data collection $a_{2}$	Data storage $a_{3}$	Demand level $a_{1}$	Scarcity level $a_{2}$	Data coverage $a_{1}$	Data timeliness $a_{2}$
$U/ind(A - \{ a\} )$	$$\begin{gathered} \{ \{ 1,3\} , \{ 5\} ,\{ 10\} , \hfill \\ \{ 2,9\} , \{ 4,7\} , \{ 6,8\} \} \end{gathered}$$	$$\begin{gathered} \{ \{ 2,9\} , \{ 4\} ,\{ 5\} , \hfill \\ \{ 1,3,10\} , \{ 6,7,8\} \} \end{gathered}$$	$$\begin{gathered} \{ \{ 1,3\} , \{ 5\} ,\{ 7\} , \hfill \\ \{ 2,4,9\} , \{ 6,8\} , \{ 10\} \} \end{gathered}$$	$$\begin{gathered} \{ \{ 7,9\} , \{ 1,2,4\} , \hfill \\ \{ 3,5,6,8,10\} \} \end{gathered}$$	$$\begin{gathered} \{ \{ 2,4\} , \{ 3,7,9\} , \hfill \\ \{ 1,5,6,8,10\} \} \end{gathered}$$	$$\begin{gathered} \{ \{ 2,3,7\} , \{ 1,5,8,10\} , \hfill \\ \{ 4,6,9\} \} \end{gathered}$$	$$\begin{gathered} \{ \{ 3,9\} , \{ 4,6\} , \hfill \\ \{ 1,2,5,7,8,10\} \} \end{gathered}$$
$I(A - \{ a\} )$	0.820	0.760	0.800	0.620	0.620	0.660	0.560
$Sig_{A} (a)$	0.020	0.080	0.040	0.140	0.140	0.080	0.180
$\omega_{A} (a)$	0.140	0.570	0.290	0.500	0.500	0.310	0.690

Considering the weights of the primary indicators, the final weights for each indicator under construction cost, data demand, and data characteristics are $\tilde{\omega }_{{A_{3} }} = [0.033,0.135,0.069]$, $\tilde{\omega }_{{A_{4} }} = [0.169,0.169]$, and $\tilde{\omega }_{{A_{5} }} = [0.132,0.293]$, respectively.

Evaluation of the participating enterprises under the original data collectors is conducted. Data cost is an objective indicator where higher costs result in higher allocation values. Therefore, using Eq. (22), the costs of Enterprise 1 and Enterprise 2 are normalized, resulting in $\tilde{f}_{1} (A_{3}^{2} ) = [0.600,0.600,0.375]$ and $\tilde{f}_{2} (A_{3}^{2} ) = [0.400,0.400,0.625]$. Furthermore, according to Eq. (24), the evaluation values for Enterprise 1 and Enterprise 2 under data cost are calculated as $L_{3}^{\prime \prime } (1) = 0.127$ and $L_{3}^{\prime \prime } (2) = 0.110$, respectively.

Data demand is a subjective indicator, and the evaluation values for the participating enterprises are determined using the fuzzy comprehensive evaluation method. The calculation process is shown in Table 20.

**Table 20:** The evaluation process of data demand indicators for enterprises participating under the original data collectors.

$d$	$R_{{4}} (d)$	$T_{{4}} (d)$	$L^{\prime}_{{4}} (d)$
Enterprise 1	$$\left[ {\begin{array}{*{20}c} {0.1} & {0.2} & {0.4} & {0.2} & {0.1} \\ 0 & {0.2} & {0.2} & {0.3} & {0.3} \\ \end{array} } \right]$$	$$\left[ {\begin{array}{*{20}c} {0.0{17}} & {0.068} & {0.101} & {0.0{85}} & {0.068} \\ \end{array} } \right]$$	0.193
Enterprise 2	$$\left[ {\begin{array}{*{20}c} 0 & {0.1} & {0.3} & {0.5} & {0.1} \\ {0.1} & {0.1} & {0.4} & {0.3} & {0.1} \\ \end{array} } \right]$$	$$\left[ {\begin{array}{*{20}c} {0.017} & {0.034} & {0.118} & {0.{135}} & {0.034} \\ \end{array} } \right]$$	0.196

The evaluation of data characteristics for the participating enterprises is conducted. The secondary indicators " data coverage " and " data timeliness " correspond to profit-related and cost-related indicators, respectively. Using Eq. (22) and (23), the normalization results for Enterprise 1 and Enterprise 2 are $\tilde{f}_{1} (A_{5}^{1} ) = [0.400,0.250]$ and $\tilde{f}_{2} (A_{5}^{1} ) = [0.600,0.750]$, respectively. Subsequently, the evaluation values are calculated as $L_{5}^{\prime \prime } (1) = 0.126$ and $L_{5}^{\prime \prime } (2) = 0.299$.

The subjective evaluation values and objective evaluation values for Enterprise 1 and Enterprise 2 under the original data collectors are obtained by summing the subjective and objective evaluation values, resulting in subjective evaluation values of $L^{1\prime } (1) = 0.193$ and $L^{1\prime } (2) = 0.196$ and objective evaluation values of $L^{1\prime \prime } (1) = 0.253$ and $L^{1\prime \prime } (2) = 0.409$. According to Eqs. (26) and (27), this study takes $\alpha = 0.5$ (the value of $\alpha$ can be adjusted according to the actual situation), resulting in normalized comprehensive evaluation values for Enterprise 1 and Enterprise 2 of $\tilde{L}^{1} (1) = 0.424$ and $\tilde{L}^{1} (2) = 0.576$. Further calculations using Eqs. (30) and (31) yield the modified revenue allocation values for Enterprise 1 and Enterprise 2 under the original data collectors as $\tilde{\varphi }_{1}^{1} (v) = 24.543$ and $\tilde{\varphi }_{2}^{1} (v) = 40.417$, respectively.

#### Revenue allocation among participants of the data processors

Using the traditional Shapley value method, the adjusted profit distribution for the data processing party is allocated to Enterprise 2, Enterprise 3, and Enterprise 4, resulting in $\varphi_{1}^{2} (v) = 1.874$, $\varphi_{2}^{2} (v) = 3.673$, and $\varphi_{3}^{2} (v) = 2.773$.

Calculate the weighting of primary indicators among participants under the data processors, with individual expert rating weights as shown in Table 21. According to Eqs. (10) and (11), the entropy weights for the primary indicators of the data processing party are calculated as $\omega_{6} = 0.630$ and $\omega_{7} = 0.370$.

**Table 21:** The weighting of primary indicator scores for the data processors.

$A$	Data cleansing $A_{6}$	Data analysis $A_{7}$
Expert 1	0.059	0.087
Expert 2	0.118	0.044
Expert 3	0	0.044
Expert 4	0.118	0.13
Expert 5	0.176	0.174
Expert 6	0.059	0
Expert 7	0.059	0.13
Expert 8	0.235	0.174
Expert 9	0.176	0.13
Expert 10	0	0.087

Determine the weights of secondary indicators for the data processors, with the classification results of expert ratings shown in Table 22. The calculation process for the secondary indicator weights is presented in Table 23. It is worth noting that the elements within the sets in Table 23 correspond to the indices of the scoring experts in Table 22.

**Table 22:** Classification results of secondary indicator scores for the data processors.

$A$	Data cleansing $A_{6}$		Data analysis $A_{7}$	
	Data volume	Data quality	Analysis methods	Analysis utility
Expert 1	1	1	3	3
Expert 2	3	2	2	2
Expert 3	2	1	1	1
Expert 4	1	3	3	3
Expert 5	2	2	2	2
Expert 6	1	1	1	1
Expert 7	2	3	3	2
Expert 8	3	2	3	3
Expert 9	2	2	3	3
Expert 10	1	3	2	1

**Table 23:** Process of calculating weights for the secondary indicators of the data processors.

$A$	Data cleansing $A_{6}$		Data analysis $A_{7}$	
$U/ind(A{)}$	$\{ \{ 1,6\} ,\{ 2,8\} ,\{ 3\} ,\{ 4,10\} ,\{ 5,9\} ,\{ 7\} \}$		$\{ \{ 1,{{4,8,9\} ,\{ 2,5\} ,\{ 3,6\} ,\{ 7\} ,\{ 10\} }}\}$	
$I(A)$	0.820		0.740	
$a$	Data volume $a_{1}$	Data quality $a_{2}$	Analysis methods $a_{1}$	Analysis utility $a_{2}$
$U/ind(A - \{ a\} )$	$$\begin{gathered} \{ \{ 1,3,6\} , \{ 2,5,8,9\} , \{ 4,7,10\} \} \end{gathered}$$	$$\begin{gathered} \{ \{ 1,4,6,10\} , \{ 2,8\} ,\{ 3,5,7,9\} \} \end{gathered}$$	$$\begin{gathered} \{ \{ 1,4,8,9\} , \{ 2,5,7\} ,\{ 3,6,10\} \} \end{gathered}$$	$$\begin{gathered} \{ \{ 1,4,7,8,9\} , \{ 2,5,10\} ,\{ 3,6\} \} \end{gathered}$$
$I(A - \{ a\} )$	0.660	0.640	0.660	0.620
$Sig_{A} (a)$	0.160	0.180	0.080	0.120
$\omega_{A} (a)$	0.470	0.530	0.400	0.600

Taking into account the entropy weights of the primary indicators, the final weights for each indicator under data cleansing and data analysis are obtained as $\tilde{\omega }_{{A_{6} }} = [0.296,0.334]$ and $\tilde{\omega }_{{A_{7} }} = [0.148,0.222]$.

The participating enterprises under the data processors are then evaluated based on the indicators. The secondary indicators under data cleansing are all objective indicators, with the evaluation for the data quality indicator based on the improvement in the proportion of data that meets the criteria of completeness, validity, and consistency compared to the original data. The normalized evaluation vectors for Enterprise 2, Enterprise 3, and Enterprise 4 under the data cleansing indicators are $\tilde{f}_{1} (A_{6}^{2} ) = [0.250,0.311]$, $\tilde{f}_{2} (A_{6}^{2} ) = [0.450,0.331]$, and $\tilde{f}_{3} (A_{6}^{2} ) = [0.300,0.358]$, respectively, resulting in evaluation values of $L_{6}^{\prime \prime } (1) = 0.178$, $L_{6}^{\prime \prime } (2) = 0.244$, and $L_{6}^{\prime \prime } (3) = 0.208$.

The data analysis situation of the participating enterprises is evaluated using fuzzy evaluation, as shown in Table 24.

**Table 24:** The evaluation process of data analysis indicators for enterprises participating under the data processors.

$d$	$R_{{7}} (d)$	$T_{{7}} (d)$	$L^{\prime}_{{7}} (d)$
Enterprise 2	$$\left[ {\begin{array}{*{20}c} {0.2} & {0.2} & {0.3} & {0.3} & 0 \\ 0 & {0.2} & {0.5} & {0.2} & {0.1} \\ \end{array} } \right]$$	$$\left[ {\begin{array}{*{20}c} {0.030} & {0.074} & {0.155} & {0.0{89}} & {0.022} \\ \end{array} } \right]$$	0.185
Enterprise 3	$$\left[ {\begin{array}{*{20}c} 0 & {0.1} & {0.6} & {0.2} & {0.1} \\ {0.1} & {0.1} & {0.4} & {0.3} & {0.1} \\ \end{array} } \right]$$	$$\left[ {\begin{array}{*{20}c} {0.022} & {0.037} & {0.178} & {0.096} & {0.037} \\ \end{array} } \right]$$	0.203
Enterprise 4	$$\left[ {\begin{array}{*{20}c} {0.1} & {0.2} & {0.4} & {0.2} & {0.1} \\ 0 & {0.2} & {0.3} & {0.3} & {0.2} \\ \end{array} } \right]$$	$$\left[ {\begin{array}{*{20}c} {0.015} & {0.074} & {0.126} & {0.096} & {0.059} \\ \end{array} } \right]$$	0.207

The subjective evaluation values for Enterprise 2, Enterprise 3, and Enterprise 4 under data analysis are $L^{2\prime } (1) = 0.185$, $L^{2\prime } (2) = 0.203$, and $L^{2\prime } (3) = 0.207$, respectively, while the objective evaluation values are $L^{2\prime \prime } (1) = 0.178$, $L^{2\prime \prime } (2) = 0.244$, and $L^{2\prime \prime } (3) = 0.208$.

By synthesizing both subjective and objective evaluation values and normalizing them, we obtain $\tilde{L}^{2} (1) = 0.296$, $\tilde{L}^{2} (2) = 0.365$, $\tilde{L}^{2} (3) = 0.339$. Consequently, the modified revenue allocation values for Enterprise 2, Enterprise 3, and Enterprise 4 under the data processors are $\tilde{\varphi }_{1}^{2} (v) = 1.564$, $\tilde{\varphi }_{2}^{2} (v) = 3.936$, $\tilde{\varphi }_{3}^{2} (v) = 2.820$.

#### Revenue allocation among participants of the data product producers

According to Eq. (4), the initial revenue allocation for Enterprise 4 and Enterprise 5 under the data product producers is calculated as $\varphi_{1}^{{3}} (v) = {9}{\text{.360}}$ and $\varphi_{{2}}^{{3}} (v) = {13}{\text{.360}}$.

Using Eqs. (7)–(11), the weights of the primary indicators under the data product producers are calculated as $\omega_{8} = 0.440$ and $\omega_{9} = 0.560$, as shown in Table 25.

**Table 25:** Process of calculating entropy weights for the primary indicators of the data product producers.

$j$	Product development $A_{8}$	Product maintenance $A_{9}$
$e_{j}$	0.919	0.897
$1 - e_{j}$	0.081	0.103
$\omega_{j}$	0.440	0.560

Experts are invited to evaluate the factors influencing the data product producers, and their ratings are used to determine the weights based on rough set theory. The classification results of the expert ratings are presented in Table 26. By applying Eqs. (13)–(16), the weights for the secondary indicators under product development are calculated as $\omega_{{A_{8} }} (a_{1} ) = 0.690$ and $\omega_{{A_{8} }} (a_{2} ) = 0.310$, while the weights for the secondary indicators under product maintenance are calculated as $\omega_{{A_{9} }} (a_{1} ) = 0.530$ and $\omega_{{A_{9} }} (a_{2} ) = 0.470$. Combining the entropy weights of the primary indicators for the data product producers, the final weights for the secondary indicators are obtained as $\tilde{\omega }_{{A_{8} }} = [0.304,0.136]$ and $\tilde{\omega }_{{A_{9} }} = [0.297,0.263]$.

**Table 26:** Classification results of secondary indicator scores for the data product producers.

$A$	Product development $A_{8}$		Product maintenance $A_{9}$	
	Workload	Difficulty coefficient	Stability	Update frequency
Expert 1	3	2	2	1
Expert 2	1	1	1	1
Expert 3	1	1	3	2
Expert 4	3	3	3	2
Expert 5	1	1	2	1
Expert 6	1	1	1	3
Expert 7	3	2	2	3
Expert 8	3	2	3	3
Expert 9	3	1	1	1
Expert 10	3	2	3	3

The normalized numerical vectors for Enterprise 4 regarding product development and product maintenance indicators are $\tilde{f}_{1} (A_{8}^{2} ) = [0.457,0.429]$ and $\tilde{f}_{1} (A_{9}^{2} ) = [0.518,0.400]$, while for Enterprise 5, they are $\tilde{f}_{2} (A_{8}^{2} ) = [0.523,0.571]$, $\tilde{f}_{2} (A_{9}^{2} ) = [0.482,0.600]$.Considering the weights of the secondary indicators, the evaluation values for Enterprise 4 under the data product producers are calculated as $L^{\prime\prime}_{8} (1) = 0.197$ and $L^{\prime\prime}_{9} (1) = 0.259$, while for Enterprise 5, they are $L^{\prime\prime}_{8} (2) = 0.237$ and $L^{\prime\prime}_{9} (2) = 0.301$. Combining the evaluation values of the two primary objective indicators yields $L^{3\prime \prime } (1) = 0.456$ and $L^{3\prime \prime } (2) = 0.538$. Since all the evaluation indicators for the data product producers are objective, the term $\alpha$ in Eq. (26) is set to 0, resulting in a normalized comprehensive evaluation value of $\tilde{L}^{3} (1) = 0.459$ and $\tilde{L}^{3} (2) = 0.531$. Therefore, the modified revenue allocation values for Enterprise 4 and Enterprise 5 under the data product producers are $\tilde{\varphi }_{1}^{3} (v) = 8.428$ and $\tilde{\varphi }_{2}^{3} (v) = 14.292$.

#### Final revenue allocation scheme for participants

After obtaining the modified revenue allocation values for each participant under each role, it is necessary to synthesize the profit values for the actual participants since there is an intersection of participants across roles. In this case, the set of all participating enterprises is denoted as $N = \{ 1,2,3,4,5\}$, and the participating enterprise sets for the original data collectors, the data processors, and the data product producers are $N_{1} = \{ 1,2\}$, $N_{2} = \{ 2,3,4\}$, and $N_{3} = \{ 4,5\}$, respectively.

The final profit values for the participating enterprises are calculated using Eqs. (36) and (37), as shown in Table 27.

**Table 27:** The final revenue allocation scheme for participating enterprises (unit: ten thousand RMB).

$x$	$\hat{\varphi }_{x}^{1} (v)$	$\hat{\varphi }_{x}^{2} (v)$	$\hat{\varphi }_{x}^{3} (v)$	$\overline{\varphi }_{x} (v)$
Enterprise 1	24.543	0	0	24.543
Enterprise 2	40.417	1.564	0	41.981
Enterprise 3	0	3.936	0	3.936
Enterprise 4	0	2.820	8.428	11.248
Enterprise 5	0	0	14.292	14.292
Total	64.96	8.32	22.72	96

## Conclusions

The circulation and trading of road data assets contribute to enhancing data value and promoting digital transportation applications. A fair and reasonable revenue allocation mechanism is key to achieving this goal. This paper proposes a two-layer revenue allocation model for road data assets based on a modified Shapley value. The model first categorizes participating companies into three roles: original data collectors, data processors, and data product producers, based on the process of realizing data value. Subsequently, a revenue allocation evaluation index system is established, considering the characteristics of different roles. At the first layer, the model allocates revenues among the three roles and introduces risk indicators for adjustment purposes. At the second layer, the model redistributes the adjusted revenues to participating companies under each role, while designing evaluation indicators specific to each role to modify the initial revenue allocation for each company. Finally, the profits of participating companies under each role are synthesized to obtain the final profit allocation for each company. This two-layer approach, combining the Shapley value with modifications, achieves a fair and effective distribution of road data asset profits.

Case studies verify that the model effectively addresses the revenue allocation issues among multiple roles in the road data asset value chain, achieving fair and reasonable allocation results. The innovation of this model lies in the role categorization and two-layer revenue allocation mechanism, which fully considers the characteristics and contributions of different roles, as well as the differences among participating companies within the same role, thereby achieving a fair and reasonable profit allocation. Specifically, the evaluation index system can be flexibly adjusted according to the actual situation. This research provides new ideas and methods for the revenue allocation of road data assets, offering important references for promoting the utilization and circulation of road data assets.

## Data Availability

The datasets used and/or analyzed during the current study are available from the corresponding author upon reasonable request.
